# Intrinsically photosensitive retinal ganglion cells in glaucoma

**DOI:** 10.3389/fncel.2022.992747

**Published:** 2022-09-23

**Authors:** Jingyi Gao, Ignacio Provencio, Xiaorong Liu

**Affiliations:** ^1^Department of Biology, University of Virginia, Charlottesville, VA, United States; ^2^Department of Ophthalmology, University of Virginia, Charlottesville, VA, United States; ^3^Program in Fundamental Neuroscience, University of Virginia, Charlottesville, VA, United States; ^4^Department of Psychology, University of Virginia, Charlottesville, VA, United States

**Keywords:** glaucoma, ipRGC, ipRGC types, type-specific degeneration, vision loss, non-visual disorders

## Abstract

Glaucoma is a group of eye diseases afflicting more than 70 million people worldwide. It is characterized by damage to retinal ganglion cells (RGCs) that ultimately leads to the death of the cells and vision loss. The diversity of RGC types has been appreciated for decades, and studies, including ours, have shown that RGCs degenerate and die in a type-specific manner in rodent models of glaucoma. The type-specific loss of RGCs results in differential damage to visual and non-visual functions. One type of RGC, the intrinsically photosensitive retinal ganglion cell (ipRGC), expressing the photopigment melanopsin, serves a broad array of non-visual responses to light. Since its discovery, six subtypes of ipRGC have been described, each contributing to various image-forming and non-image-forming functions such as circadian photoentrainment, the pupillary light reflex, the photic control of mood and sleep, and visual contrast sensitivity. We recently demonstrated a link between type-specific ipRGC survival and behavioral deficits in a mouse model of chronic ocular hypertension. This review focuses on the type-specific ipRGC degeneration and associated behavioral changes in animal models and glaucoma patients. A better understanding of how glaucomatous insult impacts the ipRGC-based circuits will have broad impacts on improving the treatment of glaucoma-associated non-visual disorders.

## Introduction

Glaucoma affects more than 70 million people worldwide and causes 10% of those afflicted to be bilaterally blind, making it one of the main causes of blindness (Quigley and Broman, 2006; Li et al., 2014). Glaucoma is characterized by retinal ganglion cell (RGC) damage resulting in subsequent vision loss. The pathogenesis of glaucoma is poorly understood, although elevated intraocular pressure (IOP) is one of the main risk factors for glaucoma (reviewed by Quigley, 2016; Artero-Castro et al., 2020). Rodent models of glaucoma have thus been developed to mimic this aspect of glaucoma using surgical methods or genetic tools to induce IOP elevation with differing severities and durations (Sappington et al., 2010; Feng et al., 2013; Thomson et al., 2014, 2020). Studies, including ours, have shown the gradual degeneration of RGCs in a cell type-dependent manner in mouse glaucoma models [reviewed by Puyang et al. (2015) and Ou et al. (2016)]. A subclass of RGCs, the intrinsically photosensitive retinal ganglion cells (ipRGCs), has drawn a lot of attention for its resistance to insult and disease (Feigl et al., 2011; Cui et al., 2015; Obara et al., 2016; Honda et al., 2019; Gao et al., 2022). The defining feature of ipRGCs is that they express melanopsin (Provencio et al., 1998, 2000, 2002). They can be classified into six types, M1 through M6, that innervate various brain targets serving a broad array of visual and non-visual responses to light [reviewed by Do (2019) and Sondereker et al. (2020)]. These responses include but are not limited to the photoentrainment of circadian rhythms (Panda et al., 2002; Ruby et al., 2002), the pupillary light reflex (Lucas et al., 2003), the photic control of mood and sleep (Ayaki et al., 2016; Maruani and Geoffroy, 2022), and the acute photic suppression of melatonin biosynthesis (Panda et al., 2003). This review is focused on type-specific ipRGC loss and the consequences on visual and non-visual effects of light with glaucoma development and progression.

### Rodent and primate intrinsically photosensitive retinal ganglion cells

Since their discovery, a plethora of studies have been published on ipRGCs, and we now know that this group of cells is heterogeneous (reviewed by Do, 2019; Lucas et al., 2020; Sondereker et al., 2020). In rodents, ipRGCs specifically labeled with melanopsin antisera have cell bodies located in both the ganglion cell layer (GCL) and inner nuclear layer (INL), with dendrites stratifying in the OFF sublamina of the inner plexiform layer (IPL) (Provencio et al., 2002; Baver et al., 2008). These cells are named M1 ipRGCs (M1s), and M1s with somata located to the INL are named displaced M1 ipRGCs (dM1s) (Berson et al., 2010; Gao et al., 2022). Retrograde tracing showed that the majority of the suprachiasmatic nucleus (SCN)-projecting RGCs are M1s and dM1s (Baver et al., 2008). M1s can be divided further into two populations based on expression of Brn3b, a transcription factor expressed in RGCs (Chen et al., 2011). The Brn3b-negative M1s innervate the SCN exclusively, while the Brn3b-positive M1s innervate other brain targets such as the olivary pretectal nucleus (OPN) (Hattar et al., 2002; Chen et al., 2011; Do, 2019). M1s are mainly involved in non-image-forming functions such as circadian photoentrainment, the pupillary light reflex, and the photic regulation of sleep (Ruby et al., 2002; Lucas et al., 2003; Panda et al., 2003; Semo et al., 2014; Do, 2019; Duda et al., 2020).

A second type of more faintly labeled ipRGC with dendrites stratifying in the ON sublamina of the IPL are named M2 ipRGCs (Baver et al., 2008). Although a small percentage of SCN innervation come from the M2s, suggesting their involvement in circadian functions, M2s also project to the dorsal lateral geniculate nucleus (dLGN), a principal target of RGCs in the thalamus that is important for image-forming vision (Ecker et al., 2010). M1s and M2s constitute a fraction of the total RGC population in mouse retina exhibiting a density of about 50–70 cells/mm^2^ [see Tables 1, 2; Berson et al. (2010); Jain et al. (2012), Hughes et al. (2013), and Gao et al. (2022)].

**TABLE 1:** Comparisons of intrinsically photosensitive retinal ganglion cell (ipRGC) densities in various models.

Species	Cell type	Quantification method	Density (cells/mm^2^)	Cells per retina	References
Mouse	Total ipRGCs	Transgene (*Opn4*^Cre/+^*; Z/AP*)		2,058 ± 141 (*n* = 4)	Ecker et al., 2010
		Immunohistochemistry	113 ± 21 (*n* = 9)	1,194 ± 281 (*n* = 9)	Jain et al., 2012
		Transgene (*Opn4.Cre ± EYFP^+/+^*)		4,415–4,705 (*n* = 2)	Hughes et al., 2013
		Immunohistochemistry		1,021 ± 109 (*n* = 19) in C57BL/6, 962 ± 169 (*n* = 21) in albino	Valiente-Soriano et al., 2014
		Immunohistochemistry	219.6 ± 3.0 (*n* = 16, ±SEM)		Gao et al., 2022
	*M1	Immunohistochemistry and Transgene (*Opn4^tau–lacZ^*)		600–780	Hattar et al. 2002; 2006
	M1	Immunohistochemistry	63	891–920	Berson et al., 2010; Hughes et al., 2013
		Immunohistochemistry	54.4 ± 3.1 (*n* = 3, ±SEM)		Gao et al., 2022
	Brn3b- M1	Immunohistochemistry	71 ± 22 (*n* = 4)	749 ± 309 (*n* = 4)	Jain et al., 2012
	Displaced M1	Immunohistochemistry	^∧^25	250	Berson et al., 2010
		Immunohistochemistry	7.0 ± 0.7 (*n* = 16, ±SEM)		Gao et al., 2022
	M2	Immunohistochemistry	59	827–830	Berson et al., 2010; Hughes et al., 2013
	*M4	Immunohistochemistry	61	850	Berson et al., 2010
	M4	Transgene (*Opn4*^Cre/+;^* Brainbow-1.0*) and Immunohistochemistry		856	Schmidt et al., 2014
		Immunohistochemistry	52.2 ± 2.0 (*n* = 10, ±SEM)		Gao et al., 2022
Rat	Total ipRGCs	Immunohistochemistry		2,320 and 2,590	Hattar et al., 2002
	Displaced ipRGCs	Immunohistochemistry		^∧^116 and 130	
Tree shrew	Total ipRGCs	Immunohistochemistry		2,899 and 3,272	Johnson et al., 2019
	M1	Immunohistochemistry	About 10	^∧^736 and 954	
	Displaced M1	Immunohistochemistry		268 and 256	
	M2	Immunohistochemistry		27 and 81	
	dopaminergic ipRGCs	Immunohistochemistry		1,868 and 1,981	
Macaque	Total ipRGCs	Immunohistochemistry	20–25 in the parafovea	About 3,000	Liao et al., 2016
Human	Total ipRGCs	Immunohistochemistry	30–30 in central retina; 8–10 in periphery	About 4,400	Liao et al., 2016
		Immunohistochemistry		7,520 and 7,046	Hannibal et al., 2017
		Immunohistochemistry	4.77	4,700	Esquiva et al., 2017
		Immunohistochemistry	2.47		Mure et al., 2019
	M1	Immunohistochemistry	0.51 ± 0.27 (*n* = 24, ±SEM) in superior nasal area		Esquiva et al., 2017
		Immunohistochemistry	15–18 in central retina; 10–11 in periphery (*n* = 4)		Nasir-Ahmad et al., 2019
	Displaced M1	Immunohistochemistry	2.05 ± 0.47 (*n* = 24, ±SEM) in superior nasal area		Esquiva et al., 2017
	M2	Immunohistochemistry	0.65 ± 0.33 (*n* = 24, ±SEM) in superior nasal area		
	M3	Immunohistochemistry	0.97 ± 0.44 (*n* = 24, ±SEM) in superior nasal area		

*Subtype classified based on morphology or quantification method which was not specified in the original study.

^∧^Estimated numbers from figures or calculated from other measurements in the study. Numbers are reported as mean ± SD if not otherwise stated.

**TABLE 2:** Intrinsically photosensitive retinal ganglion cell (ipRGC) degeneration in glaucoma.

Species	Model	Control	Glaucoma	ipRGC% decrease	Total RGC% decrease	References
Mouse	Experimental glaucoma	219.6 ± 3.0 (*n* = 16, ±SEM)	183.4 ± 4.5 cells/mm^2^ 6–9 m (*n* = 11, ±SEM)	16.5% (total ipRGCs)	32.6%	Gao et al., 2022
	Optic nerve transection	66 ± 7 cells/mm^2^ (*n* = 6, ±SEM)	^∧^About 28 cells/mm^2^	58% at 1 month (m) (*M1–M3)	88% at 1 m	Robinson and Madison, 2004
	Thy1-CFP-DBA/2J	48 ± 3 cells/mm^2^ at 2 m	19 ± 4 cells/mm^2^ at 14 m	^∧^60% at 14 m (*M1–M3)	^∧^33% at 14 m	Zhang et al., 2013
	Experimental glaucoma	1059 ± 79 and 1,019 ± 140 cells/retina from two control groups (*n* = 7)	629 ± 254 and 478 ± 248 cells/retina at 2 and 4 weeks (w, *n* = 7)	40 and 53% at 2 and 4 w (M1–M3)	^∧^34 and 59% at 2 w and 4 w	Valiente-Soriano et al., 2015b
	Transgene (GLAST)	^∧^27–29 cells/mm^2^	^∧^24–26 cells/mm^2^	NS (*M1)	48.4 ± 0.9% (±SEM)	Honda et al., 2019
	Optic nerve crush			^∧^30 and 60% at 14 and 28 d (M1); ^∧^Almost all gone at 14 and 28 d (M2)	^∧^80 and 90% at 14 days (d) and 28 d	Duan et al., 2015
	Experimental glaucoma	54.4 ± 3.1 (*n* = 3, ±SEM)	56.4 ± 1.1 cells/mm^2^ 6–9 m (*n* = 4, ±SEM)	NS (M1)	32.6%	Gao et al., 2022
		7.0 ± 0.7 (*n* = 16, ±SEM)	6.7 ± 1.0 cells/mm^2^ 6–9 m (*n* = 12, ±SEM)	NS (displaced M1)		
		52.2 ± 2.0 (*n* = 10, ±SEM)	38.8 ± 2.7 cells/mm^2^ at 6–9 m (*n* = 10, ±SEM)	25.7% (M4)		
	Experimental glaucoma	^∧^About 75 cells/mm^2^	^∧^About 55 cells/mm^2^	NS (M4)	^∧^About 66% at 14 d (transient OFF alpha RGC)	Ou et al., 2016
Rat	Experimental glaucoma	1,374 ± 74 cells/retina (*n* = 4, ±SEM)	763 ± 146 cells/retina (*n* = 4, ±SEM)	^∧^44% at 10 w (*M1–M3)	About 40% at 10 w	De Zavalía et al., 2011
	Experimental glaucoma	2,178 ± 169 cells/retina (*n* = 6)	1,082 ± 324 and 1,108 ± 255 cells/retina at 12 and 15 d (*n* = 4)	^∧^50 and 49% at 12 and 15 days (M1 and M2)	^∧^44 and 54% at 12 and 15 d	Valiente-Soriano et al., 2015a
	Experimental glaucoma	^∧^25–30 cells/mm^2^		NS (*M1)	^∧^7, 22, 28, and 24% at 2, 4, 8, and 12 w	Li et al., 2006
	Optic nerve transection			60% at 7 and 14 d (*M1–M3)	40 and 90% at 7 and 14 d	Li et al., 2008
	Optic nerve transection	2292 ± 210 cells/retina	787 ± 54 cells/retina at 6 m	66% at 6 m (*M1–M3)	99% at 6 m	Nadal-Nicolás et al., 2015
		61 ± 16 cells/retina	19 ± 8 cells/retina at 15 m	65% at 15 m (Displaced ipRGC)		
	Optic nerve crush	2,292 ± 210 cells/retina	862 ± 67 cells/retina at 6 m	65% at 6 m (*M1–M3)	96% at 6 m	
		61 ± 16 cells/retina	23 ± 7 cells/retina at 6 m	62% at 6 m (Displaced ipRGC)		
	Experimental glaucoma			63 and 61% at 14 and 45 d (*M1–M3)	75 and 87% at 14 and 45 d	Rovere et al., 2016
Human	**LHON and DOA	18, 13, and 8 cells/mm^2^ in three controls	9, 8, and 7 cells/mm^2^ in two LHON and one DOA	About 50% (total ipRGCs)	74 and 98% in two LHON, and 94% in DOA	La Morgia et al., 2010
	Glaucoma	3.08 ± 0.47 cells/mm^2^	3.00 ± 0.13 in mild and 1.09 ± 0.35 cells/mm^2^ in severe glaucoma	NS between control and mild; ^∧^About 65% in severe glaucoma (total ipRGC)	NS between control and mild; ^∧^99% in severe glaucoma	Obara et al., 2016
		^∧^About 1.4 cells/mm^2^	^∧^1.6 in mild and 0.8 cells/mm^2^ in severe glaucoma	NS (INL ipRGC)		
		^∧^About 1.7 cells/mm^2^	^∧^1.4 in mild and 0.2 cells/mm^2^ in severe glaucoma	NS between control and mild; ^∧^About 90% in severe glaucoma (GCL ipRGC)		

^∧^Estimated numbers from figures or calculated from other measurements in the study. *Subtype classified based on morphology or quantification method which was not specified in the original study.

**Leber hereditary optic neuropathy (LHON) and Kjer type dominant optic atrophy (DOA). Numbers are reported as mean ± SD if not otherwise stated; NS, not significant.

A third type of ipRGCs, the M3s (Berson et al., 2010; Ecker et al., 2010) have dendrites stratifying in both ON and OFF sublaminae of the IPL, with somata more closely resembling those of M2s than M1s in size and labeling intensity (Schmidt et al., 2011). However, the M3 population is extremely sparse, representing less than 10% of ipRGCs labeled with melanopsin antisera. Furthermore, it does not tile the retina in a regular mosaic causing some to question whether this population meets the criteria for a true RGC type (Sanes and Masland, 2015).

More ipRGC types were identified using a Cre-based knock-in mouse line that expresses Cre recombinase in place of the melanopsin gene (*Opn4*) open reading frame (Ecker et al., 2010). When crossed with a cre-dependent human placental alkaline phosphatase reporter mouse line, approximately 2,058 ± 141 cells per retina were reported to express melanopsin, a larger number than previously described using standard immunostaining methods (see Table 1). An additional ipRGC type, the M4 ipRGC, also known as the sustained ON alpha cell (Estevez et al., 2012; Schmidt et al., 2014), was labeled in this line (Ecker et al., 2010). The M4s have larger somata and dendritic arbors than the M1s, M2s, and M3s and, like the M2s, have dendrites exclusively stratifying in the ON sublamina of the IPL. Though M4s can be labeled with melanopsin antisera with or without signal amplification, they express a lower level of melanopsin (Berson et al., 2010; Estevez et al., 2012; Schmidt et al., 2014; Sonoda et al., 2020; Gao et al., 2022) and show lower intrinsic photosensitivity in single-cell electrophysiological recordings compared to M1s (Estevez et al., 2012; Hu et al., 2013). Retrograde tracing confirmed that M4s projects to the superior colliculus (SC) and dLGN, suggesting these cells are involved in image-forming vision (Estevez et al., 2012; Zhao et al., 2014; Cang et al., 2018). The density of M4s in the mouse retina is about 50 cells/mm^2^, slightly lower than the M1 and M2s (see Table 1).

The M5 ipRGC type is also labeled in Cre-based knock-in mouse lines (Ecker et al., 2010; Hughes et al., 2013; Stabio et al., 2018; Sonoda et al., 2020). M5s have compact and highly branched dendrites stratifying in the ON sublamina of the IPL. They exhibit chromatic opponency and project heavily to the dLGN and vLGN, suggesting their involvement in image-forming vision.

One last type, the M6 ipRGC, was identified in pigmented Cdh3-GFP BAC transgenic mice (Quattrochi et al., 2019). They have bistratified dendrites and small somata distinguishable from other ipRGCs. Like the M5s, they express a very low level of melanopsin and project to the dLGN, suggesting they likely contribute to the image-forming vision. As methods to selectively label these cells are limited, the density of M5s and M6s are not clear.

Intrinsically photosensitive retinal ganglion cells also have been identified in other mammals such as the tree shrew (Johnson et al., 2019), whale (Ruzafa et al., 2022), and primates (Liao et al., 2016; Esquiva et al., 2017; Hannibal et al., 2017; Mure et al., 2019; Nasir-Ahmad et al., 2019). Adult human eyes contain about 4,000–7,500 cells immunolabeled with anti-melanopsin antisera (see Table 1). Four types of ipRGCs have been identified based on morphological features. Two of the identified types correlate with the murine M1 and M2 ipRGCs. However, about half of the human M1s have their soma located to the INL instead of the GCL (Liao et al., 2016; Esquiva et al., 2017; Nasir-Ahmad et al., 2019). In another study, the human M1s were subdivided into four different subtypes: the giant M1s (GM1s) which have large somata located to the GCL and large dendritic fields, the M1s which have smaller soma in the GCL and smaller dendrites fields than the GM1s, and displaced versions of the M1s and GM1s (Hannibal et al., 2017). The intrinsic photosensitivity of human M1s has also been confirmed using multielectrode array recording (Mure et al., 2019).

The human M2s are monostratified and have their highly branched dendrites terminating in the inner border (ON sublamina) of the IPL. Their somata are located in the GCL and significantly larger than the M1 somata (Liao et al., 2016; Nasir-Ahmad et al., 2019). They are less sensitive to light compared to the M1s (Mure et al., 2019). The human counterpart of mouse M3s have bistratified dendrites and are distributed mainly in the inferior and nasal aspects of the retina (Liao et al., 2016; Hannibal et al., 2017; Nasir-Ahmad et al., 2019). In one study, however, M3s were found to form a regular mosaic (Esquiva et al., 2017). Finally, the human M4s, have dendrites located in the ON sublamina of the IPL (Hannibal et al., 2017), though at a slightly greater depth compared to the M2s, similar to that observed in the mouse retina (Estevez et al., 2012). To date, no human M5 or M6 ipRGCs have been identified, which may be due to the limited access to samples and inadequate sensitivity of the techniques used to label ipRGCs.

Central targets of ipRGCs have been described in macaque (Hannibal et al., 2014). Using anterograde tracing and pituitary adenylate cyclase-activating polypeptide (PACAP) immunostaining, Hannibal et al. (2014) found that macaque ipRGCs project to the SCN, LGN, SC, and other brain areas. It is likely that like the mouse ipRGCs, primate ipRGCs contribute to both image-forming and non-image-forming vision. More studies are needed to elucidate how each type of primate ipRGCs contributes to the variety of functions.

### Non-uniform distribution of intrinsically photosensitive retinal ganglion cell subtypes

Studies are inconsistent on whether ipRGC distribution is uniform across the retina (Hughes et al., 2013; Honda et al., 2019; Quattrochi et al., 2019; Sonoda et al., 2020). In rat, preferential distribution of ipRGCs is found in the dorsal retina (Vugler et al., 2008). Hughes et al. (2013) showed that total ipRGCs (M1 to M5) labeled in the *Opn4^Cre±^;EYFP^+/+^* mouse line exhibited a uniform density, but M1 and M2 ipRGC types labeled by melanopsin antisera showed a superior-inferior gradient with almost one-fold difference. The M4 and M5 cells collectively exhibited the reverse gradient, where significantly higher density was found in the inferior retina (Hughes et al., 2013). In other two studies, M4 cells exhibit highest density in the mouse temporal-superior retina, with a reduction in dendritic arbor sizes (Bleckert et al., 2014; Sonoda et al., 2020).

The distribution of ipRGCs also varies across the retina of the tree shrew *Tupaia belangeri*, a protoprimate, where ipRGCs show highest density in the ventral-temporal retina (Johnson et al., 2019). Moreover, different ipRGC types have different spatial arrangement; while the bistratified ipRGCs were highest in the temporal retina, the dopaminergic ipRGCs were more uniformly distributed. In human retina, ipRGC density and dendritic morphology also differ depending on retinal location (Hannibal et al., 2017; Nasir-Ahmad et al., 2019). While M1s show the highest density in the temporal retina, GM1s, M2s and M4s are more densely distributed in the nasal retina. In fact, M2s and M4s are absent in the peripheral region of the temporal retina (Hannibal et al., 2017). The higher density of M2 cells in the nasal retina was confirmed by another study (Nasir-Ahmad et al., 2019).

The differential distribution of human ipRGCs may have biological significance as localized illumination of different regions of the retina shows different effectiveness in melatonin suppression (Glickman et al., 2003; Rüger et al., 2005). In mice, the opsin gradients in the retina correlate with the spectral tuning of ipRGC types (Hughes et al., 2013). Alternatively, mouse strain differences could account for the non-uniform distribution of ipRGCs. The total number of ipRGCs between albino Swiss and C57BL/6 mice are similar, but ipRGCs are more densely distributed in the temporal retina in C57BL/6, while they are more abundant in the superior retina of albino Swiss mice (Valiente-Soriano et al., 2014). More work is needed to characterize the spatial distribution of ipRGCs and the attendant behavioral functions, which will build a solid baseline for studying ipRGC density change in diseased conditions.

## Intrinsically photosensitive retinal ganglion cell degeneration in glaucoma

The survival of ipRGCs has been examined in various rodent glaucoma models and in glaucoma patients, but results remain equivocal (Li et al., 2006; Drouyer et al., 2008; La Morgia et al., 2010; De Zavalía et al., 2011; Feigl et al., 2011; Kankipati et al., 2011; Wang et al., 2013; Gracitelli et al., 2014; Duan et al., 2015; Kelbsch et al., 2016; Kuze et al., 2017; Vidal-Sanz et al., 2017; Ciulla et al., 2019; Ahmadi et al., 2020; Gao et al., 2022). Many studies suggest that ipRGCs suffer less degeneration than the general RGC population in animal glaucoma models (Li et al., 2006; De Sevilla Müller et al., 2014; Ou et al., 2016; Rovere et al., 2016; Gao et al., 2022; see Table 2). For example, Li et al. (2006) induced continuous IOP elevation for four months through laser photocoagulation of the episcleral veins of rat eyes. They found that although the general SC-projecting RGCs suffered about a 24% cell loss, M1 ipRGC density and dendritic architecture remained stable (Li et al., 2006). Similarly, more than 30% of rat ipRGCs survived, while only 25 and 13% of pan RGCs remained at 14 and 45 days, respectively, after a 75 min acute ocular hypertension (Rovere et al., 2016). In our recently published study, mild IOP elevation was induced for more than 3 months in the mouse retina by laser photocoagulation of the trabecular meshwork (Gao et al., 2022). At 6–8 months post-laser surgery, the ipRGCs labeled by melanopsin antisera suffered 16% cell loss, while the general population of RGCs suffered a significantly greater 25–32% cell loss (Gao et al., 2022; see Table 2). In addition to RGC soma loss, reduction in RGC dendritic field size and decrease in dendrite complexity are also observed in glaucoma (Della Santina et al., 2013; Feng et al., 2013; El-Danaf and Huberman, 2015; Bhandari et al., 2019). Della Santina et al. (2013) showed that OFF-transient RGCs exhibited decreased dendritic coverage, dendritic length, and number of dendrites. At 6–8 weeks post IOP elevation, we found that the dendritic coverage of mono-laminated ON but not bi-laminated ON-OFF cells decreased (Feng et al., 2013). Specifically, a subtype of ON cells, the SMI-32-positive ON cells, showed significantly reduced dendritic branching (Feng et al., 2013). Interestingly, studies suggest that the M1 ipRGC dendrites undergo expansion post optic nerve crush (ONC) and in dystrophic retina (Vugler et al., 2008; De Sevilla Müller et al., 2014). Overall, these studies are in agreement that ipRGCs may be less vulnerable in response to the disease insults.

However, some rodent studies suggest that ipRGCs degenerate at a similar rate, if not a faster than the general RGC population in glaucoma (see Table 2). In rats, short-term IOP elevation was induced for about 2 weeks and ipRGCs were found to degenerate substantially, similar to the general RGC population (Valiente-Soriano et al., 2015a). When IOP elevation was induced for 10 weeks by injection of chondroitin sulfate into the anterior chamber of the rat eyes, a significant 50% reduction in melanopsin expression was found, while the expression level of the general RGC marker Thy-1 suffered a comparable 45% reduction (De Zavalía et al., 2011). This result, however, could be interpreted as that melanopsin protein expression itself fluctuates following IOP elevation (Nadal-Nicolás et al., 2015; Vidal-Sanz et al., 2017). Another study showed that the general RGCs suffered a 33% cell loss, while the ipRGCs (likely M1s) suffered a significantly greater 60% cell loss at 14 months of age of Thy1-CFP-DBA/2J mice, a genetic model of glaucoma (Table 2; Zhang et al., 2013). Greater reductions in dendritic arbor and complexity of ON alpha cells (M4s) were also observed compared to OFF alpha cells in ocular hypertension models and in DBA/2J mice (Risner et al., 2018).

The different disease phenotypes observed in the above studies may be due to type-specific changes within ipRGC population, and/or the variability in the severity and duration of IOP elevation induced in the different models of glaucoma. For example, we showed that M4s and M2s suffered a 25 and 23% cell loss, but the density of M1s and displaced M1s remained stable (Gao et al., 2022). In other studies, the M4s, also known as ON sustained alpha cells, are found to preferentially survive compared to the OFF and ON-OFF alpha cells (Ou et al., 2016) and panRGCs (De Sevilla Müller et al., 2014; Bhandari et al., 2019). Vidal-Sanz et al. (2017) found that panRGCs labeled by Brn3a degenerate in two phases: an acute phase with fast degeneration followed by a phase with much slower but continuous cell loss. ipRGCs also degenerated quickly in the acute phase, but then reached a plateau with no further cell loss, thus leading to a higher survival rate compared to panRGCs (Rovere et al., 2016; Vidal-Sanz et al., 2017). Such pattern has also been observed in M1s by two research groups (Li et al., 2008; De Sevilla Müller et al., 2014).

It is possible that a higher IOP elevation is required to cause observable morphological damage to ipRGCs. In studies where IOP is mildly elevated by less than two-fold, M1s tend to suffer little or no degeneration (Li et al., 2006; Ou et al., 2016; Gao et al., 2022). When IOP is greatly elevated > 1.5- to three-fold, significant cell loss of ipRGCs is observed (Valiente-Soriano et al., 2015a,b; Rovere et al., 2016). The duration of IOP elevation may also affect how ipRGCs behave in different glaucoma models. In DBA/2J mice, ipRGCs seem to be more resistant to degeneration than panRGCs at 5 months of age, when IOP is just starting to increase. But they become more vulnerable and suffer larger degeneration than pan RGCs at 14 months of age, when IOP has been continuously and significantly elevated (Zhang et al., 2013). Future studies looking at how the extent and duration of IOP elevation affects type dependent RGC degeneration is needed. While variations in study design may obscure a generalized conclusion of ipRGC degeneration in the context of glaucoma, the ongoing research may offer insights for clinical studies where patients also suffering from varied degrees of IOP elevation exhibit varied rates of deterioration of visual and non-visual functions.

Studies that directly address ipRGC survival in human glaucoma cases are lacking. To date, only one group examined ipRGCs survival in human glaucoma patients and age-matched controls (Obara et al., 2016). They found that while almost all RGCs labeled by the pan RGC marker rbpms (Rodriguez et al., 2014) were gone in severe glaucoma patients, ipRGCs only suffered about a 50% loss. Interestingly, while ipRGCs in the GCL suffered a substantial 88% cell loss, no significant cell loss was found in the ipRGCs with somata in the INL, suggesting human displaced M1s cells are more resistant to glaucomatous damage than non-displaced M1s. Another study examined the cell loss within the general RGC population and ipRGCs in post-mortem retinas from patients with Leber hereditary optic neuropathy (LHON) and Kjer type dominant optic atrophy (DOA) (La Morgia et al., 2010). They found that although most RGCs have degenerated in the three patients examined ranging from a 74 to 98% loss, only about half of the ipRGCs were lost (see Table 2).

In fact, it seems possible to examine ipRGC survival in glaucoma patients indirectly. Employing a receptor silent substitution paradigm which works at the phototransduction-cascade level of vision by controlling the effective quantal catch of photoreceptors, one study compared the electrophysiological responses of ipRGCs in glaucoma patients to normal subjects; and they found that the amplitude of ipRGC responses of glaucoma patients was greatly reduced compared to those of the controls (Kuze et al., 2017). As glaucomatous human retinas are difficult to acquire and study, proto-primate models of glaucoma have been explored (Burgoyne, 2015). For example, an experimental tree shrew glaucoma model has been developed that induces IOP elevation through microbead injection into the eye (Samuels et al., 2018). Three ipRGC subtypes have been identified in the tree shrew retina that share morphological traits with the primate ipRGCs (Johnson et al., 2019). These studies may offer insights on how primate/human ipRGCs respond to the glaucoma insult.

## Various mechanisms contribute to intrinsically photosensitive retinal ganglion cell resistance to glaucomatous damage

The underlying mechanisms of RGC loss remain poorly understood. One hypothesis is that elevated IOP can cause mechanical stress on the lamina cribrosa beneath the optic nerve head (ONH), where the axons bundles exit the eye (Quigley et al., 1981; Bellezza et al., 2000; Levin, 2001; Chidlow et al., 2011; Calkins, 2012; Weinreb et al., 2014). This stress leads to disruption of axonal transport, as well as disorganization of microtubules and neurofilaments at the ONH, causing metabolic stress for the RGCs (Burgoyne, 2011; Calkins, 2012; Howell et al., 2013). These initial insults to axons eventually are transmitted to the somata, leading to apoptotic degeneration of the RGC cell bodies (Howell et al., 2013; Quigley, 2016).

The optic nerve crush (ONC) and optic nerve transection (ONT) models have been used to study rapid RGC degeneration. ipRGCs have been found to survive better than general RGCs in ONC and ONT models (Robinson and Madison, 2004; De Sevilla Müller et al., 2014; Duan et al., 2015; Vidal-Sanz et al., 2017; Tran et al., 2019). In mice, panRGCs labeled by Tuj1 suffered 88% cell loss while ipRGCs (likely M1s) suffered only a 58% cell loss at one month post-ONT (Robinson and Madison, 2004). Results from mouse ONC models also agreed with the results from the rat ONT model. While the general RGC population suffered 80 and 90% cell loss at 14- and 28-days post-ONC, respectively (see Table 2), M1s suffered only 30 and 60% cell loss, respectively (Duan et al., 2015). The M4s demonstrated even higher survival rates than the M1s, with only 20 and 40% cell loss, respectively. The M2s, on the other hand, were almost all absent (Duan et al., 2015). The same results were confirmed in another study using the single cell RNA-seq techniques (Tran et al., 2019). All ipRGCs were found to be resistant to degeneration at 14-days post-ONC, with M4s showing the highest survival rate among all ipRGCs (Tran et al., 2019).

Though an elevated IOP is associated with a majority of glaucoma cases, some patients do not exhibit elevated IOP (Weinreb et al., 2014), suggesting that factors other than IOP elevation contribute to glaucoma disease progression (Calkins, 2012; Wang et al., 2020). Some glaucoma patients show downregulation of glutamate transporters (Naskar et al., 2000). The GLAST KO mouse model is devoid of a transporter of aspartate and glutamate and exhibits progressive loss of RGCs and axon bundles in the absence IOP elevation (Honda et al., 2019). In these mice, when half of the general RGC population had degenerated, only 10% of ipRGCs (M1s and M2s) were lost. Moreover, alpha RGCs, a group that contains M4s, also showed excellent resistance to degeneration, suffering only a 4% cell loss.

Indeed, many factors may contribute to ipRGC survival in glaucoma. In various axonopathies where axons degenerate in a manner whereby they die back, longer axons tend to suffer more damage or die more quickly than shorter ones, possibly due to higher metabolic demands (Stassart et al., 2018). While the majority of the RGC involved in image-forming visual functions innervate the superior colliculus and dLGN, the Brn3b negative M1 cells innervate the SCN, a region much closer to the ONH (Baver et al., 2008; Chen et al., 2011; Duan et al., 2015; Sanes and Masland, 2015). As a dying back mechanism is responsible for some axon degeneration in glaucoma (Crish et al., 2010), the shorter routes of the M1 axons may be protective. In addition, M1s are also known to send axon collaterals to regions inside of the retina such as the ciliary marginal zone (Semo et al., 2014) and the nerve fiber layer (Joo et al., 2013). These collaterals may provide protection for those M1 cells; as observed in mouse spinal injury, a surviving intact branch of axon suppresses retrograde degeneration of the injured branch (Lorenzana et al., 2015). Future studies are needed to investigate the subtype-dependent ipRGC axonal damage and their underlying mechanisms in glaucoma.

Intrinsically photosensitive retinal ganglion cells may also receive more trophic support than other RGC types in a diseased condition. Neurotrophic factors (NTFs) are diffusible molecules that bind tropomyosin related kinase (Trk) receptors and p75 receptors (p75NTR) [reviewed by Huang and Reichardt (2003)]. RGCs receive NTFs from two sources: (1) target-derived factors secreted from higher brain centers innervated by RGC axons, and (2) local trophic factors that are produced by retinal neurons and glial cells. Target-derived trophic factors are transported along the RGC axon retrogradely and hindered axonal transport of target-derived NTFs may contribute to RGC apoptosis. Interestingly, ipRGCs, especially M1s, may be able to receive target-derived NTFs from their axon collaterals within the eye, which offers an alternative route for NTFs (Joo et al., 2013; Semo et al., 2014). In addition, the intrinsic photosensitivity of ipRGCs allows them to initiate neuronal activity without receiving inputs from other retinal interneurons. Compared with artificial supplementation of NTFs, neuronal activity may induce more physiologically relevant NTFs and receptor expression in the neurons (Corredor and Goldberg, 2009). In glaucoma, dendrites of panRGCs tend to shrink in size and complexity (Feng et al., 2013; Puyang et al., 2015), leading to a reduction in inputs and altered neuronal activity. ipRGCs, on the other hand, may retain robust intrinsic activity with light stimulation. For example, as discussed above, in ocular hypertensive mice, M1 dendrites undergo remodeling and expansion which in turn may be beneficial for their survival (Li et al., 2006; Vugler et al., 2008; De Sevilla Müller et al., 2014). Endogenous NTFs, secreted by Müller glial cells, such as BDNF, ciliary neurotrophic factors (CNTF), and insulin like growth factor (IGF), are also upregulated upon electrical stimulation in cultured retina (Sato et al., 2008; Manthey et al., 2017). However, some studies suggest that the endogenous NTFs may offer little long-term protection compared to target-derived NTFs. Following the ONC injury, the application of BDNF both to the eye and brain showed long-term protection of cat RGCs, while application to the eye only temporarily rescued RGCs (Weber et al., 2010).

Studies have begun to characterize the downstream signaling pathways of ipRGC survival in glaucoma. For example, ipRGCs maintain a relatively high level of mTOR activity, which is associated with axon regeneration (Duan et al., 2015; Li et al., 2016). This high mTOR activity is also associated with melanopsin GPCR signaling, as ectopic expression of melanopsin in RGCs stimulated axonal regeneration (Li et al., 2016). Another study showed that the ipRGCs’ resistance to damage is reduced upon application of PI3 K/Akt specific inhibitors after ONT and ocular hypertension in rats (Li et al., 2008). These studies may offer potential drug targeting site specifically for promoting ipRGC survival and function of glaucoma patients in future.

## Intrinsically photosensitive retinal ganglion cell-related behavioral changes in glaucoma

It is known that the majority of retinal afferents to the SCN, arises from M1 ipRGCs (Baver et al., 2008), suggesting they contribute to circadian photoentrainment (Jones et al., 2015). Indeed, it has been shown that Brn3b-negative M1s are sufficient for circadian photoentrainment (Chen et al., 2011). When ipRGCs projecting to non-SCN and non-IGL (intergeniculate leaflet) brain targets are ablated (Rupp et al., 2019), mice retained circadian photoentrainment, while other functions, such as the pupillary light reflex and contrast sensitivity, showed deficits. Moreover, the M1 electrophysiological profiles are tuned to transmit accurate visual signals to the SCN (Stinchcombe et al., 2021). Therefore, M1 cell loss may lead to deficits in photoentrainment, which has been reported in several studies using rodent glaucoma models (Drouyer et al., 2008; De Zavalía et al., 2011). Using anterograde CTB tracing, one study investigated the projection of RGC axons to various brain targets and found significantly reduced CTB accumulation in the SCN of a rat model of glaucoma (Drouyer et al., 2008), which may reflect impeded axonal transport (Crish et al., 2010; Smith et al., 2016) or a loss of retinal fibers afferent to the SCN. While they retain the ability to re-entrain to shifted light-dark (LD) cycles, the number of days required to establish a stable phase of activity was significantly longer than the control rats. Moreover, the stability of the phase of activity onset was somewhat variable relative to the start of the dark period (Drouyer et al., 2008). Similarly, another group found reduced melanopsin expression and reduced ipRGC density after IOP elevation in rats that were able to entrain to shifted LD cycle, but with a delayed phase angle of activity onset (De Zavalía et al., 2011).

As degeneration of ipRGCs including M1s are also observed in human glaucoma patients (Obara et al., 2016) and in optic neuropathy (La Morgia et al., 2010), dysregulation of circadian rhythmicity was seen in primary open angle glaucoma (POAG) patients. Higher sleep disturbances, lower average sleep time, lower sleep efficiency and higher arousal are observed in glaucoma patients (Wang et al., 2013; Gracitelli et al., 2015) and the ratio of patients with sleep disorders increases with greater visual field impairment (Wang et al., 2013; Ayaki et al., 2016). Normally, light stimulation suppresses melatonin production, a process that heavily relies on ipRGCs. However, in glaucomatous mice, blue light exposure is unable to reduce pineal melatonin levels (De Zavalía et al., 2011). Decreased melatonin secretion was also seen in human glaucoma patients (La Morgia et al., 2010; Ma et al., 2018; Yoshikawa et al., 2020). Moreover, in an advanced glaucoma group, no significant nocturnal melatonin suppression is apparent after bright light exposure (Pérez-Rico et al., 2010). Together these studies suggest a possible positive correlation between M1 cell loss and non-visual deficits in glaucoma.

In normal tension glaucoma (NTG) patients, sleep quality as measured by the Pittsburgh Sleep Quality Index did not differ significantly from controls (Ahmadi et al., 2020). This phenotype may be due to the varied severity of the disease in the subjects selected. In our recently published study (Gao et al., 2022), we found that murine M1s are resistant to degeneration after sustained mild IOP elevation. However, while no significant deficit in circadian re-entrainment is seen in bilaterally lasered mice, re-entrainment is accelerated when these mice are subjected to a 6-h phase advance of a relatively bright LD cycle (Gao et al., 2022). Mice subjected to a shift of a dimmer LD cycle do not show this accelerated re-entrainment. Acceleration of re-entrainment to a shift in a bright LD cycle reflects a change in basic underlying parameters of the circadian system in the glaucomatous mice. The SCN is a hierarchical structure that maintains and regulates circadian rhythmicity through the coordination of its many autonomous cellular oscillators (Antle and Silver, 2005; Paul et al., 2009; Welsh et al., 2010). The desynchrony between these cellular oscillators may lead to a change in the rate of circadian entrainment. For example, administration of vasoactive intestinal peptide (VIP) before an LD cycle shift increases desynchrony among the individual cellular oscillators, resulting in a dampening of the amplitude of the composite SCN rhythm. This reduced amplitude facilitates the perturbation of phase necessary for re-entrainment. As such, the more dampened the amplitude, the more quickly the SCN can re-entrain its phase to a shifted LD cycle (An et al., 2013). Therefore, we hypothesize that reduced or changed inputs to the SCN, either through the alteration of the direct photic input from the retina or changes in inputs from other nuclei in the brain, may cause desynchrony between these oscillators and alter their phase distribution, ultimately leading to the subtle changes in re-entrainment that we observed in our surgical glaucoma model. Even though the M1 cell density did not change in our glaucoma mice (Gao et al., 2022), the synaptic input from them could be reduced or even enhanced. For example, impeded axonal transport and/or reduced ipRGC axonal inputs to SCN has been observed in a rat glaucoma model (Drouyer et al., 2008). Arbor shrinkage (Zhang et al., 2013) and change in dendritic complexity (El-Danaf and Huberman, 2015) have also been noticed in mice models of glaucoma. Interestingly, in aging dystrophic rat retina, the ipRGC cell count is reduced, although the total number of melanopsin-positive dendritic processes remains the same, suggesting a compensatory remodeling and expansion of the ipRGC dendrites in disease (Vugler et al., 2008). In addition, the expression level of melanopsin has been found to decrease or fluctuate in glaucoma animal models (De Zavalía et al., 2011; Vidal-Sanz et al., 2017). This may affect circadian entrainment, as melanopsin-null animals exhibit reduced phase-shifting responses (Panda et al., 2002; Ruby et al., 2002; Wang et al., 2022). Taken together, changes in ipRGC morphology, function, and axonal projections may exert subtle effects on the direct photic input to the SCN.

Glaucoma may also affect the master circadian clock through indirect pathways as the SCN also receives afferents from multiple regions in the brain (Abrahamson and Moore, 2001; Yuan et al., 2018). Specifically, Grippo et al. (2017) found that dopaminergic neurons in the ventral tegmental area (VTA) project to the SCN and their activation accelerates photoentrainment in mice (Grippo et al., 2017). A recent study found that the VTA of mice receives inputs from the preoptic area that in turn receives direct inputs from M1s (Zhang et al., 2021), a pathway involved in the regulation of non-rapid eye movement (NREM) sleep. Therefore, change in M1 axonal inputs within this pathway also indirectly may alter circadian rhythms in mice. Alternatively, we observed a decrease in M4 cells in sustained ocular hypertensive mice (Gao et al., 2022) and these M4 cells may project to the intergeniculate leaflet (IGL) (Ecker et al., 2010). The IGL encodes irradiance and sends a projection to the SCN *via* the geniculohypothalamic tract (Morin and Studholme, 2014; Yuan et al., 2018). Therefore, the degeneration of M4 cells could affect the IGL’s modulation of the retinal input to the SCN and indirectly impact its behavioral output (Hanna et al., 2017). It is important to point out that circadian dysfunction in glaucoma patients may not be solely due to ipRGC degeneration and altered photic input. Other factors, such as reduced social interactions (Golombek and Rosenstein, 2010) and mood disorders (Vadnie and McClung, 2017) may also contribute to the process.

Alterations in circadian rhythmicity are also related to hyperactivity disorders such as Attention-Deficit/Hyperactivity Disorder (ADHD) (Baird et al., 2011; Coogan et al., 2016). Therefore, similar phenotypes may exist in glaucoma. In fact, a significantly higher activity-to-rest ratio relative to controls is observed in an experimental rat model of glaucoma (De Zavalía et al., 2011) and in a genetic mouse model of glaucoma (Zhang et al., 2013). Similarly, in glaucoma patients, a significant increase in daily activity and wake time is observed (Lanzani et al., 2012). Circadian dysfunction can also be at the root of mood disorders and depression (Golombek and Rosenstein, 2010). It is becoming more appreciated that light is heavily involved in mood regulation, a process mediated mainly through ipRGCs, through SCN-dependent and independent pathways (An et al., 2020; Maruani and Geoffroy, 2022). The prevalence of mood disorder and depression is significantly higher in glaucoma patients than matched controls (Ayaki et al., 2016; Yoshikawa et al., 2020).

Intrinsically photosensitive retinal ganglion cells are also involved in other non-image-forming visual functions that are affected in glaucoma. Brn3b-positive M1 ipRGCs project to OPN (Baver et al., 2008; Ecker et al., 2010; Rupp et al., 2019), a region in the brain that controls the pupillary light reflex (PLR), the constriction of the pupil in response to bright light stimulation. In a rat model of unilateral glaucoma, with 50% ipRGC loss, a significant decrease in the magnitude of the consensual pupil constriction is observed in the contralateral intact eye (De Zavalía et al., 2011). Deficits in the PLR in human glaucoma patients have also been observed and are correlated with the severity of disease. Multiple studies have reported a significantly reduced PLR response in POAG (Nissen et al., 2014; Gracitelli et al., 2015; Kelbsch et al., 2016), NTG (Ahmadi et al., 2020), and hereditary optic neuropathy (Kawasaki et al., 2014) patients compared to healthy subjects. This reduction in PLR response is more apparent after blue light stimulation compared to red light stimulation (Nissen et al., 2014; Kelbsch et al., 2016), which is consistent with the blue light sensitivity of melanopsin, the photopigment of ipRGCs. Relative maintenance of the PLR is also seen in LHON and DOA patients (La Morgia et al., 2010). A significant difference is found in the post-illumination pupil response between advanced glaucoma and early glaucoma patients, but the deficit is not apparent between early glaucoma patients and healthy controls (Feigl and Zele, 2014), a pattern also observed in hereditary optic neuropathy (Kawasaki et al., 2014). Indeed, the degree of visual field loss is linked to the half-max intensity of pupil responses (Kawasaki et al., 2014). Interestingly, in one patient with advanced secondary glaucoma, a minimal pupillary response was retained even though no cognitive perception of light remained (Zhou et al., 2014). This is no surprise given the injury resistance of ipRGCs. Some ipRGCs may survive and function well, even in severe or late stage of glaucoma when other RGCs have almost completely degenerated.

Though a great number of ipRGC studies focus on the non-image-forming aspects of vision, increasing attention is being directed to the contributions of ipRGCs to image-forming vision. M2, M4, M5, and M6 cells and a small percentage of M1 cells send their axons to the dLGN, the principal relay for visual information to the visual cortex (Schmidt et al., 2014; Stabio et al., 2018; Do, 2019; Quattrochi et al., 2019). Such circuitry suggests that these types of ipRGCs contribute to image-forming vision. Indeed, following ablation of several ipRGC types including the M4s (Schmidt et al., 2014), contrast sensitivity is significantly reduced at various spatial frequencies (0.05 and 0.09 c/d) to which M4s respond most strongly in isolated retina (Estevez et al., 2012). We also showed that the ocular hypertensive mice exhibited a significant decrease in contrast sensitivity at 0.103 and 0.192 c/d, which correlates with M4 degeneration (Gao et al., 2022). However, this does not exclude the possibility that degeneration of other non-M4 RGCs may contribute to the change in contrast sensitivity in the OHT mice, as the general RGC population suffered substantial cell loss (Baden et al., 2016; Khani and Gollisch, 2017). Distinguishing the contributions of ipRGCs and non-ipRGC ganglion cells to image-forming vision is challenging, and much work is needed to investigate how image-forming visual functions mediated by ipRGCs are affected in glaucoma patients.

In conclusion, ipRGC-mediated image-forming and non-image-forming visual circuits and behaviors are affected in glaucoma. A better understanding of ipRGC type-dependent degeneration and the corresponding ipRGC circuits that account for the behavioral changes is needed. The results will help to improving patient care and treatment with glaucoma development and progression in the future.

## Author contributions

All authors listed have made a substantial, direct, and intellectual contribution to the work, and approved it for publication.
